# How scale affects N_2_O emissions in heterogeneous fields of a diversified agricultural landscape

**DOI:** 10.1038/s41598-025-95630-6

**Published:** 2025-03-31

**Authors:** Isabel Zentgraf, Maire Holz, Oscar Rodrigo Monzón Díaz, Matthias Lück, Katja Kramp, Valerie Pusch, Kathrin Grahmann, Mathias Hoffmann

**Affiliations:** 1 Working Group of Isotope Biogeochemistry and Gas Fluxes, Leibniz Centre for Agricultural Landscape Research (ZALF) e.V., Eberswalder Straße 84, 15374 Müncheberg, Germany; 2 Thaer-Institute of Agricultural and Horticultural Sciences, Humboldt-Universität zu Berlin , Invalidenstraße 42, 10099 Berlin, Germany; 3 Working Group of Root Soil Interaction, Leibniz Centre for Agricultural Landscape Research (ZALF) e.V., Eberswalder Sraße 84, 15374 Müncheberg, Germany; 4 Experimental Station Müncheberg, Leibniz Centre for Agricultural Landscape Research (ZALF) e.V., Eberswalder Straße 84, 15374 Müncheberg, Germany; 5 Working Group of Resource-Efficient Cropping Systems, Leibniz Centre for Agricultural Landscape Research (ZALF) e.V., Eberswalder Straße 84, 15374 Müncheberg, Germany

**Keywords:** Soil heterogeneity, N_2_O emission, Coarse-textured soils, Crop rotation, Element cycles, Environmental impact, Biogeochemistry, Environmental sciences

## Abstract

Nitrous oxide (N_2_O) emissions from agricultural soils vary due to factors such as soil organic matter, soil moisture, and crop type, leading to short-term variations and concentrated zones of high emissions, known as “hot moments” and “hotspots.” These peaks, occurring at various scales, contribute significantly to total N_2_O emissions. This is particularly relevant for sandy soils, where high porosity and low water-holding capacity promote gas diffusion and create moisture variability, leading to highly heterogeneous N_2_O emissions. We investigated N_2_O fluxes along a transect in six agriculturally used patches (0.52 ha) with varying texture, yield potential and crop rotation. We measured N_2_O fluxes bi-weekly over 2 years, using a non-flow-through non-steady-state (NFT-NSS) manual closed chamber system, covering different crops and weather conditions. Hot moments accounted for 6–71% of total crop N_2_O emissions and were mostly driven by soil physical properties. On a small scale, soil texture and environment determined spatial heterogeneity of N_2_O emissions being more pronounced for sandier soils. On patch level, N_2_O emissions differed more strongly than on microplot level and were mainly driven by crop-type and management. Our findings highlight the importance of accounting for intrinsic variability in soil texture, topography, and microclimate within patches. Additionally, broader differences across management-influenced patches must be considered to better understand the drivers of N_2_O emissions. This dual-scale approach emphasizes the need for high-resolution soil monitoring for mitigation strategies and to refine models. At the same time, it guides farmers toward soil-specific fertilization to reduce emissions and maintain yields in diverse agricultural landscapes.

**Supplementary Information:**

The online version contains supplementary material available at 10.1038/s41598-025-95630-6.

## Introduction

Nitrous oxide (N_2_O) is a potent greenhouse gas (GHG), and its warming potential is 298 times higher than that of carbon dioxide (CO_2_)^1^. With 60%, agriculture is one of the largest contributors to total N_2_O emissions^2,3^. A key driver for N_2_O emissions is the type and amount of nitrogen (N) fertilizer used. While N fertilizers are essential for achieving high crop yields, they also increase the risk of N leaching and atmospheric N losses. Studies have shown that there is a linear^4,5^ or even exponential^6^ relationship between N fertilization rates and N_2_O emissions. This is, the fertilizer N use efficiency of crops ranges around 30–50% and commonly decreases with increased N fertilization rates^7^. The remaining fertilizer is prone to losses through various pathways, such as N_2_O or NH_3_ emissions, or leaching as nitrate (NO_3_⁻)^8^.

N_2_O emissions from agricultural soils are affected by a variety of environmental factors including soil organic matter, pH, soil moisture and crop type^9–11^. These factors can create short term or small-scale variations in N_2_O emissions, often leading to periods and concentrated zones of high emissions, commonly referred to as “hot moments” and “hotspots”^12–15^. This is particularly the case for extremely sandy soils, having unique physical and chemical properties that influence nutrient dynamics and gas emissions. For instance, their coarse texture results in high porosity and generally low water-holding capacity, which promoting gas diffusion and can create localized areas of differing moisture levels. Previous research found highest N_2_O emissions occurring in most sandy, freely drained soils, when compared to less sandy, imperfectly drained soils, because increased nitrification at lower water filled pore space (WFPS) levels is facilitated by improved aeration and oxygen availability in coarse-textured soils^16,17^. In addition, sandy soils are often more vulnerable to climate change effects, such as increased precipitation variability and drought^18^. Understanding how these variations in soil properties and environmental factors affect N_2_O emissions can help to reduce GHG emissions and adapt agricultural practices in the face of changing climate conditions.

This is especially important given that temporal and spatial peaks in N_2_O emission account for a significant portion of total N_2_O emissions and can occur across different scales. Studies have documented substantial variability in N_2_O emissions across scales ranging from microscale to regional scale, with coefficients of variation between 42 and 217%^19–21^. Research also indicates that the factors driving N_2_O emissions vary depending on scale. At small-scales (< 1 m^2^), emissions are primarily linked to the presence of denitrifying microsites within the soil^22^. In contrast, at larger scales (> 10 m^2^), emissions are more strongly associated with the availability of mineral N and topographic influences rather than the physical properties of the soil^23^. Given this significant variation in driving factors across different scales, a thorough understanding of the scale dependency on N_2_O emissions is crucial for effectively managing and mitigating these N losses.

Precision agriculture and site-specific management approaches aim to reduce these N losses by tailoring practices to present spatial and temporal variability^24^. In aim of this, the “patchCROP” experiment by the Leibniz Centre for Agricultural Landscape Research (ZALF) employs a patch-based design (72 m × 72 m; 0.5 ha) to explore the effects of diverse management practices on agroecosystem responses, including GHG emissions^25^. This experimental design is particularly relevant in sandy soils, where even minor heterogeneities in soil texture and environmental conditions strongly influence N_2_O dynamics. The 70-ha experimental area represents typical field sizes found in eastern Germany as well as large parts of Eastern Europe, where socialist-era land reforms have resulted in fields up to 100 ha, contrasting with Western European fields that average 3 to 8 ha due to historical land-use patterns and smaller farm structures. These in average larger fields naturally tend to exhibit higher heterogeneity, making the patchCROP design especially suited to capturing the interaction of these drivers across spatially extensive areas.

While it is well-established that different crops and management practices result in variability in N_2_O emissions, our study goes further by linking spatial variability between patches to both management-specific and environmental drivers. Patch-level heterogeneity provides critical insights into how diverse management strategies interact across spatially extensive fields, offering a valuable framework for understanding cumulative emissions and identifying potential management and soil heterogeneity driven hotspots for N_2_O emissions. By bridging the gap between small-scale variability and broader field-scale dynamics, gained data might help to enhance accuracy of process based models and support the development of adaptive and targeted mitigation strategies for diversified agricultural systems.

To investigate N_2_O emissions and their small- and larger-scale variability across single fields characterized by pronounced spatial heterogeneities, our study adopts a multi-driver approach. This approach emphasizes the importance of integrating multiple factors—soil properties, management practices, and weather variability—into experimental designs. Traditional randomized block experiments often oversimplify real-world complexity, as they are conducted under homogeneous conditions that fail to account for the complex interactions and small-scale variations present in real agricultural fields, where soil texture, soil moisture, temperature, and other environmental factors are highly heterogeneous^26^. By using a transect-based design, our study seeks to address this gap and provide a more comprehensive understanding of N_2_O emissions in actual agricultural settings. As highlighted by Thomas & Rayan (2024) such multi-driver experiments minimize prediction error while maximizing the information obtained, making them crucial for understanding the interplay of environmental and management factors in N_2_O emissions and their spatial variability.

Building on this knowledge, the following hypotheses were tested in our study:

1. Hot moments, such as fertilization and heavy rainfall, contribute significantly to N_2_O emissions, with these events accounting for 30–70% of total seasonal emissions in rain-fed systems.
2. In sandy soils, small-scale heterogeneities in the silt-to-sand ratio influence N_2_Oemissions, with higher sand content amplifying spatial heterogeneity due to higher gas diffusion, drainage and lower water retention capability.
3. In heterogeneous fields, N_2_O emissions exhibit substantial spatial variability, driven by a continuum of interactions between soil texture, management practices, and weather conditions.

## Results

### Environmental conditions and soil properties

**Fig. 1:**
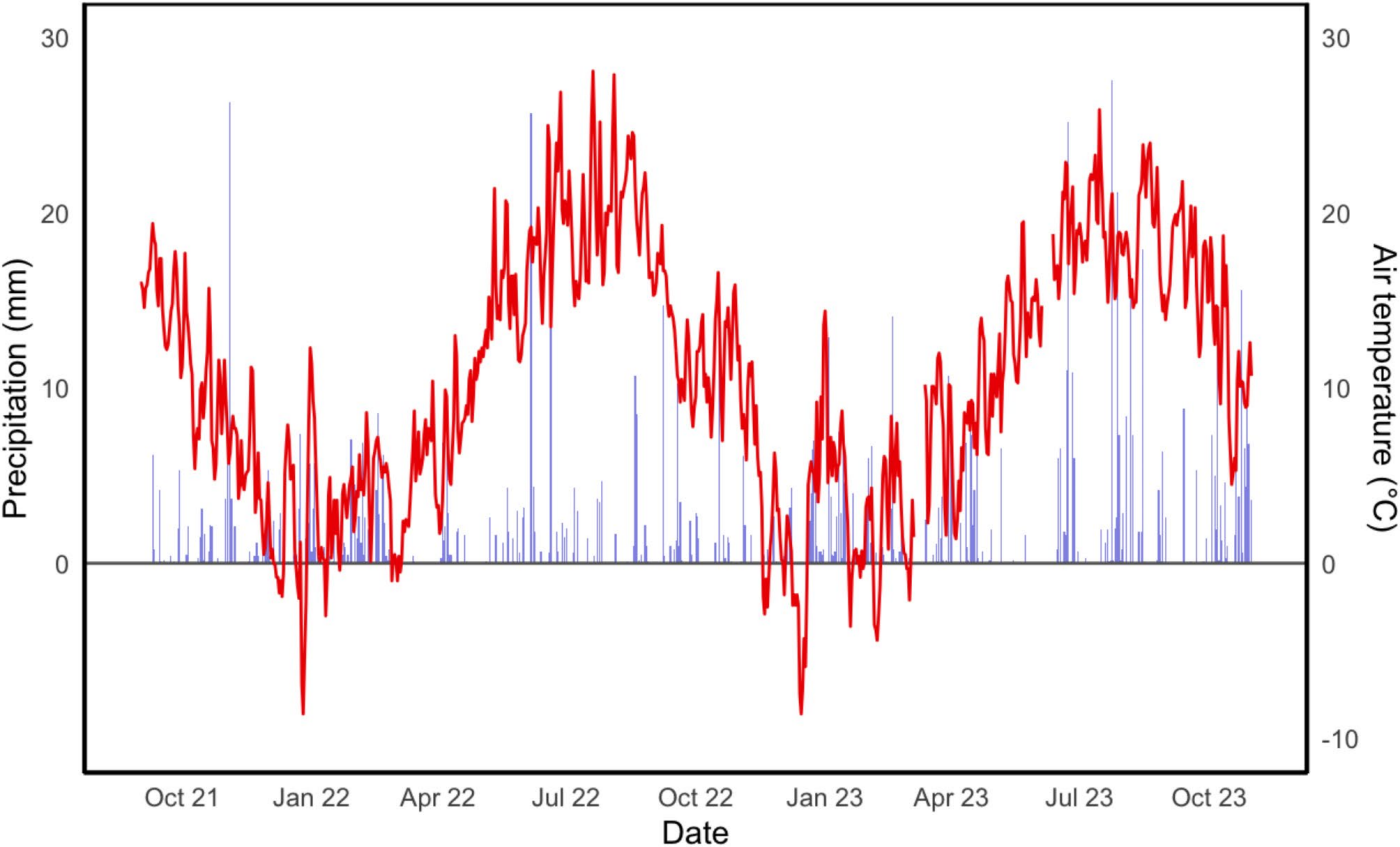
Environmental conditions at the study site throughout the study period. The red line indicates daily air temperature (°C) and blue bars daily precipitation (mm).

With an annual precipitation of 362 mm and 656 mm, respectively, 2022 was rather dry while 2023 was relatively wet compared to the long-term average of 509 mm (ZALF weather station, 2010–2024^28^). Summer months (June – August) of 2023 were exceptionally wet with a total of 210 mm compared to a total of 106 mm for June to August in 2022. Lowest monthly precipitation in 2022 was recorded for March (0.5 mm) and highest for February (57.7 mm). In 2023 lowest precipitation was in May (8.4 mm) and highest in December (90.6 mm) (Fig. 1). During the research period a total of five heavy rain events (> 20 mm d^−1^) occurred on 11/04/2021, 06/06/2022, 06/23/2023, 07/24/2023 and 07/28/2023.

With a mean temperature of 19.7 °C, the summer of 2022 was warmer than in 2023 (18.6 °C). Monthly mean air temperature ranged from 1.2 °C (December) to 20.9 °C (August) in 2022 and from 2.6 °C (February) to 19.3 °C (July) in 2023.

Soil properties of the six patches are shown in Table 1. Specific values for each microplot can be found in the supplementary table S2. Texture as well as C/N varied according to high yield and low yield classification of the respective patch with mean of 69.9% and 80.9% and 11.3 and 10.1, respectively. Highest sand content was found in patch 95 microplot four (94.5%) and lowest in patch 74 microplot five (67.6%). Soil texture was classified as sandy loam (high yield patches) and loamy sand (low yield patches). Highest total carbon (TC) and total nitrogen (TN) concentrations were found in patch 65 microplot 4 (0.99 and 0.09%, respectively), whereas lowest concentrations were found in patch 89 microplot 2 (0.59 and 0.06%, respectively). Soil pH was lower in the low yield patches compared to the high yield patches with lowest value for patch 96 (pH 5.4) and highest for patch 73 (pH 6.4). Bulk density (BD) of the upper soil (0–34 cm) was determined in spring 2021 (patches 65 and 96) and spring 2022 (patches 73, 74, 89 and 95) and ranged from 1.70 g/cm^3^ in patch 89 to 1.77 g/cm^3^ in patch 73.

**Table 1:** Soil properties of each study patch (0–30 cm).

	Patch	Sand (%)	Silt (%)	Clay (%)	pH (–)	C/N (–)	TC (%)	TN (%)	BD (g/m^3^)
High yield	65	71.0	20.5	8.5	6.0	12.3	0.9	0.1	1.73
		± 1.2	± 0.7	± 0.6	± 0.3	± 0.3	± 0.05	± 0.005	
	73	70.0	23.4	6.6	6.4	11.1	0.9	0.1	1.77
		± 1.2	± 0.8	± 1.3	± 0.1	± 0.2	± 0.06	± 0.007	
	74	68.6	24.7	6.7	6.1	10.5	0.8	0.1	1.72
		± 0.8	± 0.8	± 0.8	± 0.1	± 0.2	± 0.08	± 0.007	
Low yield	89	77.7	17.9	4.4	5.7	9.3	0.7	0.1	1.70
		± 3.2	± 2.9	± 0.6	± 0.2	± 0.4	± 0.05	± 0.006	
	95	83.3	11.8	4.9	6.0	9.9	0.7	0.1	1.73
		± 1.2	± 0.9	± 0.4	± 0.3	± 0.5	± 0.02	± 0.004	
	96	81.9	13.6	4.6	5.4	11.0	0.8	0.1	1.72
		± 1.7	± 0.9	± 0.9	± 0..2	± 0.4	± 0.07	± 0.004	

Values shown are averages per patch ± sd (n = 6).

### Temporal N_2_O flux dynamics

**Fig. 2:**
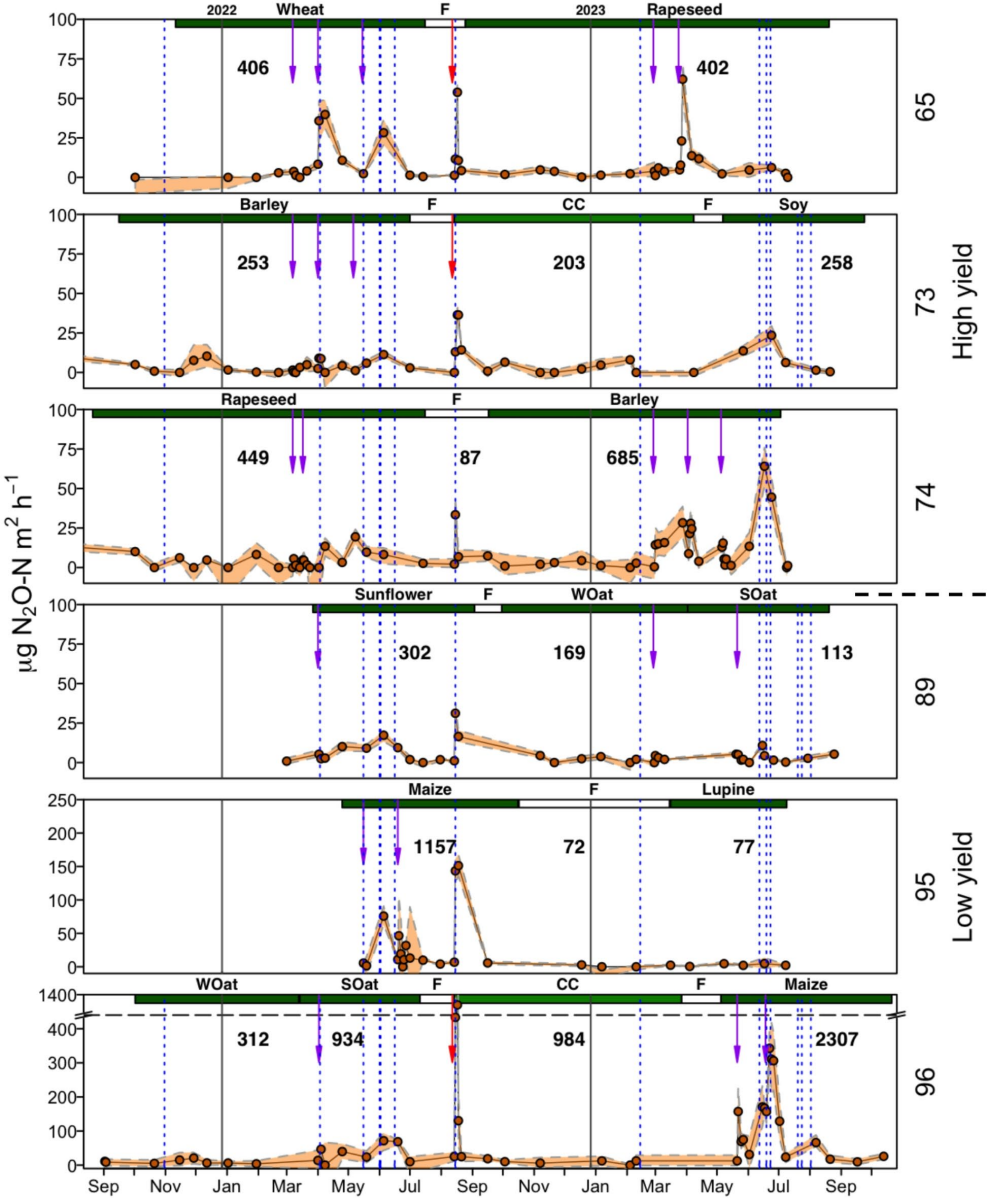
Temporal N_2_O flux dynamics of the six patches. Note that limits of y-axes for patches 95 and 96 were adjusted to 250 and 1400 µg N_2_O-N m^2^ h^−1^ respectively, to be able to show fertilization peaks. Purple arrows indicate mineral N-fertilization events, red arrows organic fertilization (digestate) and dotted blue lines substantial rain events. In bold letters the rounded cumulative N_2_O-N in g ha^−1^ crop^−1^ is displayed.

In total, more than 2.000 single N_2_O fluxes were measured during the study period. Figure 2 presents the temporal variation of the N_2_O fluxes averaged for each patch, between September 2021 and October 2023. Following mineral and organic fertilization events, indicated with purple and red arrows, respectively, increases in N_2_O fluxes were observed. The highest peaks in N_2_O emissions during the study period were observed after organic fertilizer application in patches 65, 73 and 96. Increasing N_2_O fluxes were also obtained following heavy rain events (blue dotted lines). This was especially the case towards the end of the cropping season (e.g. barley patch 74; maize patch 95). In general, time periods directly following fertilization and heavy rain events, significantly contributed to total N_2_O crop emissions, accounting for around 36% and 43% of the annual crop N_2_O emissions in 2022 and 2023, respectively (table S3).

Highest N_2_O flux dynamics and crop related N_2_O emissions were observed for maize on patch 95 (1157 g N_2_O-N ha^−1^ crop^−1^) in 2022 and on patch 96 (2307 g N_2_O-N ha^−1^ crop^−1^) in 2023. Lowest flux dynamics and crop related N_2_O emissions were obtained for the non-fertilized lupine on patch 95 and fertilized summer oat on patch 89 in 2023 (77 and 113 g N_2_O-N ha^−1^ crop^−1^, respectively).

In general, fluxes showed higher amplitudes for high yield potential patches when compared to low yield patches. Mean values for low yield vs. high yield crop emissions were 643 g N_2_O-N ha^−1^ crop^−1^ and 305 g N_2_O-N ha^−1^ crop^−1^, respectively. Based on the Wilcoxon Rank-Sum Test, N_2_O fluxes were slightly but not significant (*p* = 0.51) higher in 2023 compared to 2022 (mean of 640 and 444 g N_2_O-N ha^−1^ crop^−1^, sd = 846 and 371, respectively).

### Spatial heterogeneity of N_2_O emissions

**Fig. 3:**
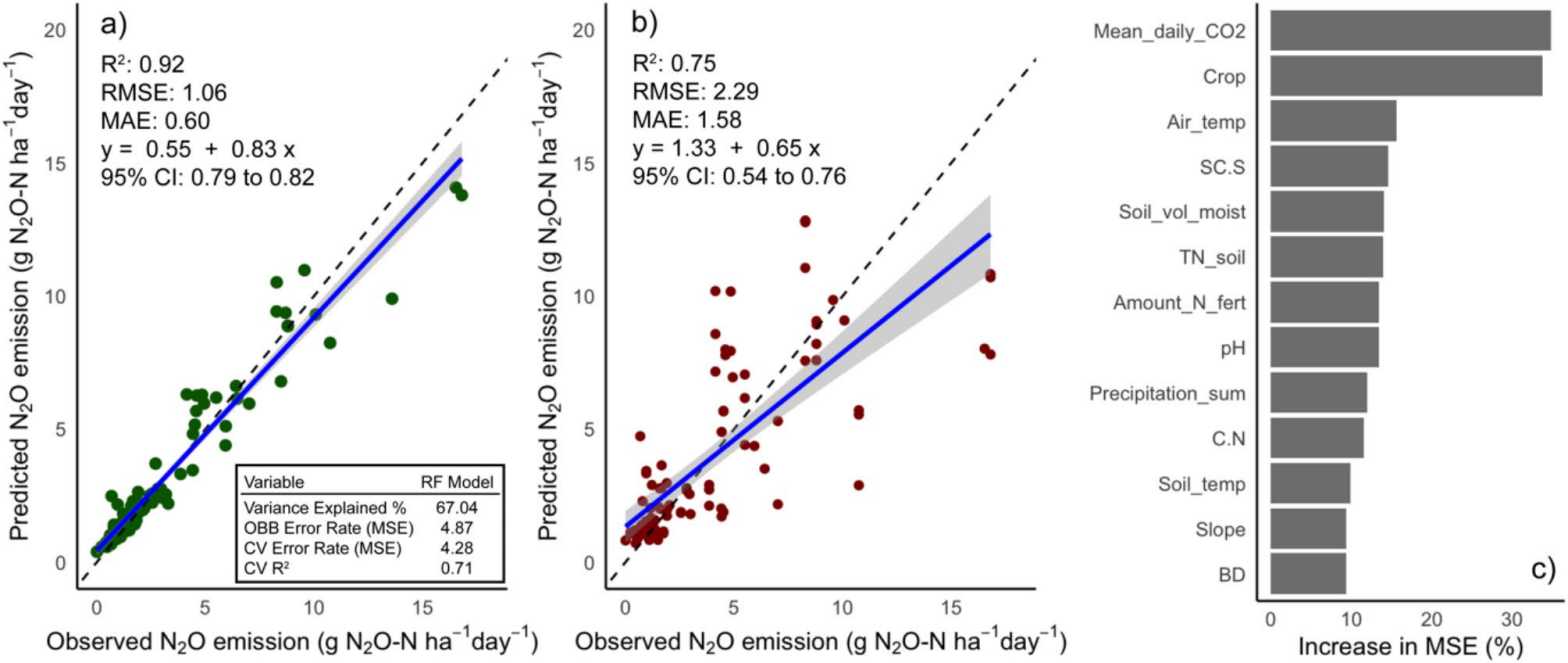
Output of the RF model aiming to explain variations in mean daily N_2_O-N emissions of each micro plot. (**a**) Model validation of the full dataset is displayed with a 1:1 line as reference to the regression line. R^2^, RMSE and MAE, 95% confidence interval (CI) and regression equation (y) from the regression of actual vs. predicted values are displayed. Specific variables of the RF model are displayed in the black box. (**b**) Predictive RF model using tenfold cross validation is displayed with a 1:1 line as reference to regression line. (**c**) Variables of importance are plotted against their increase in MSE (%) value for the RF model.

With a R^2^ value of 0.92 and an explained variance of 67%, the employed RF model was used to examine cumulative, crop related N_2_O-N emissions, demonstrating a high level of examination accuracy (Fig. 3a). The model evaluated 102 cumulative emissions, which were gap-filled using the 2.000 measured N_2_O fluxes. Also, the prediction model (tenfold cross-validation; Fig. 3b) demonstrated a strong predictive capability by achieving a R^2^ value of 0.75, a root mean squared error (RMSE) of 2.29, and a mean absolute error (MAE) of 1.58. These results demonstrate that the model was able to effectively capture and predict the variability of the N_2_O emissions in our study.

According to the variable of importance analysis (Fig. 3c), the most significant predictors of the RF model are crop type and mean daily CO_2_ emission, each contributing substantially to the MSE when permuted (> 30%). These key variables are followed by air temperature, soil texture (SC:S ratio), volumetric soil moisture content, soil total N content and amount of N fertilizer. Variables of least importance were BD and slope, each contributing less than 10% to the MSE increase.

**Fig. 4:**
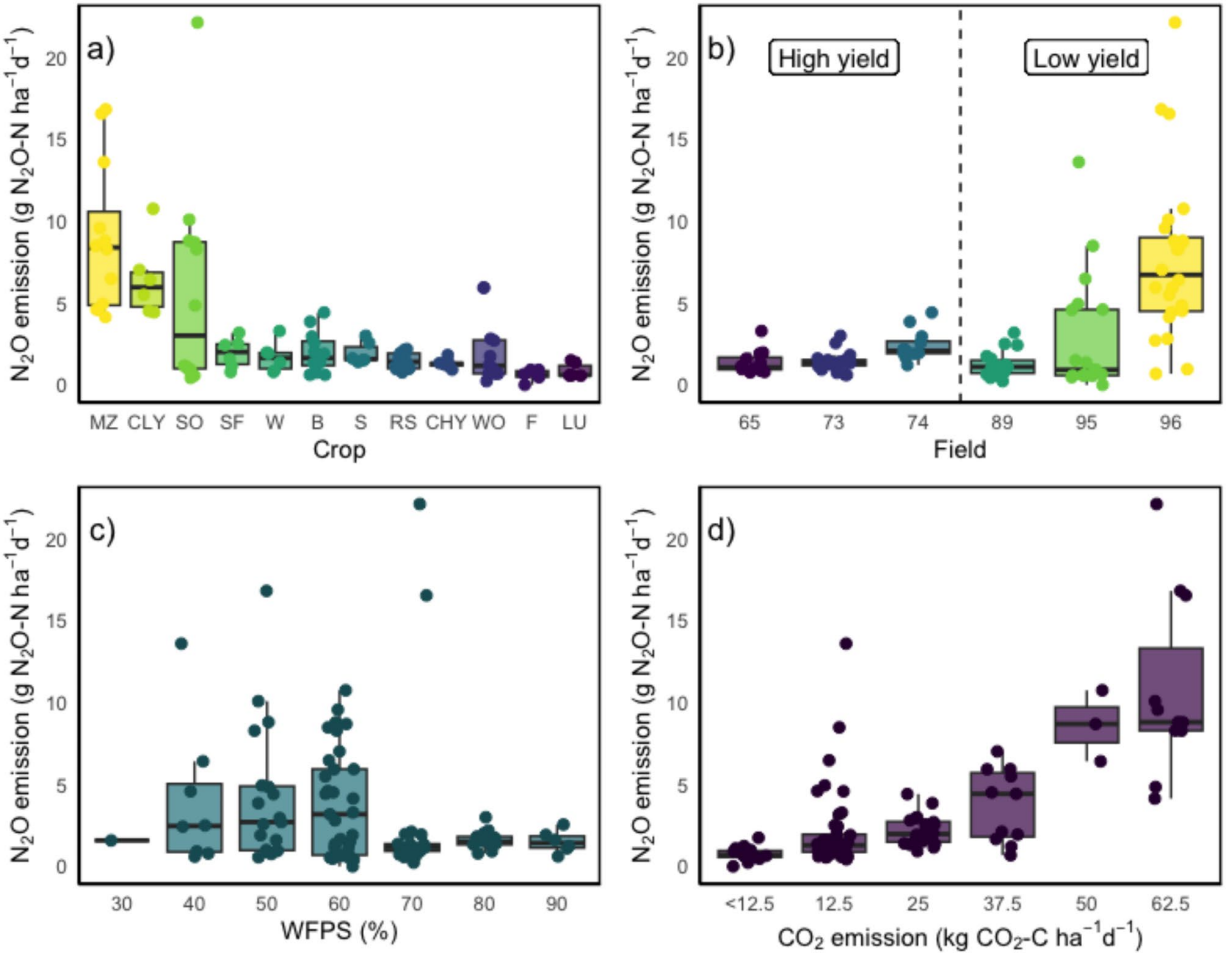
Factors explaining mean daily N_2_O emissions. (**a**) Factor crop in decreasing order. MZ, maize; CLY, cover crop low yield; SO, summer oat; SF, sunflower; W, winter wheat; B, barley; S, Soy; RS, rapeseed; CHY, cover crop high yield; WO, winter oat; F, fallow; LU, lupine. (**b**) Factor patch separated by yield. (**c**) Factor volumetric soil moisture calculated as water filled pore space (WFPS) in percentage. (**d**) Mean daily CO_2_ emission plotted against mean daily N_2_O emission.

Figure 4 shows the relation between major factors revealed by the RF and the 102 cumulative, crop related N_2_O-N emissions. First, as indicated by the RF model, a significant variation of N_2_O-N emissions was observed across different crop types (Fig. 4a). The highest N_2_O emission was found for patches planted with maize (average N_2_O emission of 8.9 g ha-1 day-1), followed by cover crop (6.4 g ha^−1^ day^−1^). Lowest N_2_O-N was emitted for patches planted with lupine (0.9 g ha^−1^ day^−1^). High yield patches emitted on average 1.7 g N_2_O-N ha^−1^ day^−1^, whereas low yield potential patches emitted 4.3 g N_2_O-N ha^−1^ day^−1^ (Fig. 4b).

Second, the strong positive correlation (R^2^ = 0.63; Fig. 4d) between mean daily CO2 and N_2_O emissions indicates that higher CO_2_ levels are associated with increased N_2_O emissions. This relationship was consistent across all patches. Highest CO_2_ emissions were found for patch 96 (56.9 kg C ha^−1^ crop^−1^) and lowest values for patch 95 (7.96 kg C ha^−1^ crop^−1^). High yield patches had a mean CO_2_ level of 19.3 kg ha^−1^ crop^−1^, whereas low yield patches had a level of 28.1 kg ha^−1^ crop^−1^. Crop specific CO_2_ levels were detected with lowest values for fallow (3.6 kg ha^−1^ crop^−1^) and highest for maize (45.8 kg ha^−1^ crop^−1^).

Third, the mean air temperature during the growing period of the specific crop was evaluated as 3^rd^ most important variable explaining N_2_O-N emissions. According to the ALE plot (Fig. S1), lower air temperatures between 4 and 12 °C had a minimal impact on the mean daily N_2_O emissions. Starting from 12 to 16 °C, we noticed a moderate increase indicating an increase in N_2_O emissions. Temperature values above 16 °C resulted in a steep increase, suggesting a strong positive relationship between mean daily N_2_O and air temperature at this higher temperature range.

Fourth, the RF model evaluated the mean volumetric soil moisture content as the 5^th^ most important variable explaining N_2_O-N emissions. In Fig. 4c, the calculated mean daily WFPS % is plotted against the mean daily N_2_O emissions. Patch and yield specific trends were observed with high yield patches showing tendentially higher mean WFPS than low yield patches (67.6% and 58.6%, respectively). The WFPS favoring N_2_O-N emissions within our patches was located between a WFPS of 40–60%. Higher WFPS > 60% values did not lead to an increase in N_2_O emissions.

At the microplot scale N_2_O emissions exhibited light to moderate correlations with soil environmental parameters like texture, soil moisture, and soil temperature. The strongest relationships were observed with silt and sand content (r = -0.36 and 0.35, respectively; *p* < 0.05), while the weakest correlation was found with WFPS (r = -0.22; *p* < 0.05) (Fig. S2).

**Fig. 5:**
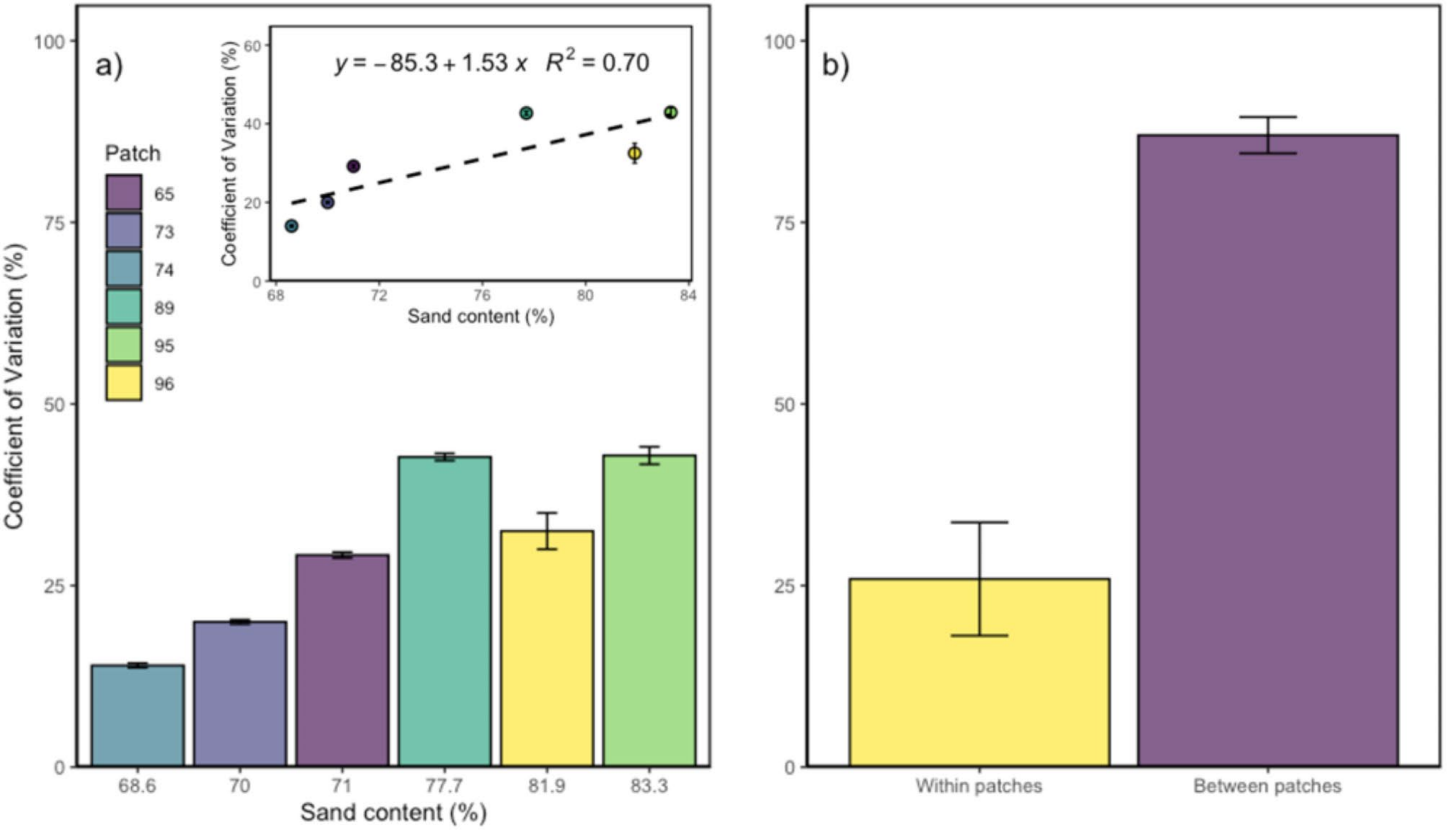
Coefficient of variation (CV) in percentage. (**a**) CV (%) per patch, ordered by increasing sand content (%) with regression plot. (**b**) CV (%) within patches and between patches.

Spatial heterogeneity in N_2_O emissions was high and present across the microplots of each patch and strongly pronounced between patches (Fig. 5a, b). The CV within patches showed a positive correlation with increasing sand content, achieving an R^2^ value of 0.7 (Fig. 5a). Levene’s test for homogeneity of variances revealed that this variation was significant (*p* < 0.05) within patches 73, 89, and 96. In contrast, patches 65, 74, and 95 did not exhibit significant differences in variability when compared to the remaining patches. Highest CV was found for patch 95 (low yield) with a value of 42.9%, whereas lowest CV was found for patch 74 (high yield) with a value of 14.0%. The average CV within all high yield patches was 21.1%, compared to 39.3% for low yield patches.

## Discussion

In our first hypothesis, we proposed that hot moments, such as fertilization and heavy rainfall, contribute significantly to N_2_O emissions from agricultural fields, accounting for 30–70% of total crop emissions in rain-fed systems. Previous research has reported that the first three months after fertilization events can account for 74–96% of total annual N_2_O emissions^29^ and that the fertilization event alone can account for up to 70% of total annual N_2_O emissions^14^. However, Krichels & Yang, (2019) showed that the spatiotemporal heterogeneity in soil N_2_O emissions is not always characterized by predictable hot moments and that controlling factors change depending on environmental conditions, which underlines the importance of understanding the driving factors for effectively mitigating N_2_O emissions.

In our study, hot moments were responsible for between 6 and 71% of the total crop specific N_2_O emissions (tab. S3), confirming our first hypothesis. Overall, hot moments were found to contribute to a larger share of total crop N_2_O emissions in “high-emission” crops like maize and barley compared to “low-emission” crops such as summer oat or lupine. While in some cases, hot moments accounted for as little as 6% of emissions, this was typically when baseline emissions were low as well. However, when substantial emissions occurred (> 500 g ha^−1^ crop^−1^), trigger events were responsible for 50 to 70% of the total crop related emissions.

In addition, we found that higher shares of hot moments appeared for summer crops like maize and soy (71 and 52%, respectively), compared to winter crops like winter oat and rapeseed (6 and 18%, respectively). This can likely be explained by higher air temperatures during trigger events for summer cropping periods. Previous studies found that warm and wet climate conditions foster N_2_O from soil, which, in general, are more favorable conditions for nitrification^11,31^. Additionally, hot spots in N_2_O emissions are particularly likely to occur following wetting events after dry periods^32,33^. The soil drying and rewetting is inducing N mineralization and denitrification, increasing N_2_O emission. This was pronounced in our study, as most heavy rain induced hot moments occurred in summer months between May and August (Fig. 2) with a corresponding WFPS of e.g. 45 and 28% before and 65 and 94% after the rain event, respectively (patch 96, maize season 2023). However, it needs to be considered that, we focused on sandy soils in a continental climate in our study, representing large areas of the northern hemisphere. When comparing sandy soils across different climates, such as continental versus maritime climates, the patterns and timing of hot moments and trigger events may differ, resulting in varying N_2_O emission dynamics.

In summary, we found that hot moments played a disproportionately large role when baseline N_2_O emissions were high compared to periods of overall low emissions. Importantly, our findings provide novel insights by demonstrating how the magnitude of hot moments shifts based on both crop type and small-scale spatial differences. For example, a single heavy rainfall event contributed more significantly to N_2_O emissions in maize compared to lupine, soy, or rapeseed (Fig. 2, June 2023), highlighting the interaction between crop-specific management practices and environmental conditions. While previous studies have emphasized the importance of event-based N_2_O measurements^12,13,15^, our results extend this understanding by showing that these peak events are not static but vary significantly in their contribution across spatial scales and crop types. This nuanced understanding underscores that peak events not only drive short-term N_2_O flux variability but also exert a greater impact under conditions of elevated baseline emissions. By focusing on these shifting emission peaks, we can refine strategies for GHG mitigation, tailoring them to specific crops, spatial characteristics, and dynamic emission patterns in arable fields.

Our second hypothesis stated that in sandy soils, small-scale differences in the silt-to-sand ratio influence N_2_O emissions, with higher sand content amplifying spatial heterogeneity due to higher gas diffusion, drainage and lower water retention capability^18^. This hypothesis was confirmed by our study.

We found that N_2_O emissions displayed high heterogeneities on a small-scale between microplots and between patches, which were more pronounced in more sandy soil (R^2^ of 0.7) (Fig. 5a, b). According to e.g. Li et al. (2022) the coarse texture can create localized areas of differing moisture levels, which can lead to a higher heterogeneity in N_2_O emissions. Previous research has also shown that soils with higher sand content and good drainage tend to exhibit elevated N_2_O emissions^16,17^, which is supporting our study. Here, we observed the greatest N_2_O-N losses in sandy soils with highest sand content, and an optimal WFPS range of 40–60% (Fig. 4b). This can be attributed to increased nitrification at lower WFPS (%) due to better aeration and oxygen availability in coarse-textured soils. These results emphasize the importance of adjusting fertilization to address the spatial variability in soil texture and moisture levels to mitigate N_2_O emissions.

Additionally, the high macro porosity of sandy soils promotes gas diffusion, creating an optimal balance for microbial activities and gas exchange. Previous research assumed that little N_2_O emissions result from denitrification in coarse textured soils^35,36^. However, we observed N_2_O emissions occurring up to a WFPS of 60%. Thus, it is likely that while oxygen started to become limited, denitrification was initiated but the denitrification process was incomplete resulting in high N_2_O emissions. A higher WFPS of > 60% did not lead to an increase in N_2_O emissions, which can be attributed to anaerobic conditions, which presumably led to a complete denitrification process and subsequent emission as N_2_.

Recent research has shown that at small scales (up to 10 m^2^), N_2_O emissions exhibit considerable heterogeneity, with CV reaching as high as 217%^21^. However, studies have shown that this variability tends to increase as the spatial scale expands^19,22^. Our findings are consistent with these results, as we observed a significantly lower heterogeneity within individual patches compared to between patches. This outcome was likely due to the intense variations in crops and management practices across the patches. Additionally, the comparable lower texture variability within individual patches may also explain the lower N_2_O variability observed within patches compared to between patches (table S2).

For our third hypothesis, we proposed that in larger fields, N_2_O emissions exhibit substantial spatial variability, driven by a continuum of interactions between soil texture, management practices, and weather conditions. This hypothesis was supported by our RF analysis which identified crop type, CO_2_ emissions and air temperature as the most significant predictors of mean daily N_2_O emissions (Fig. 3c).

Tallec et al. (2022) emphasized the role of cropping systems for N_2_O emissions, attributing observed differences to variations in N fertilization and management practices. They observed lower N_2_O emissions during winter cropping due to the split-application of smaller N amounts during well-developed vegetation growth throughout the growing season. Contrarily, higher N_2_O emissions occurred during summer cropping, driven by larger N inputs during low vegetation development in May and medium development in June. This is partially consistent with our study. Although N fertilizer amounts were site-specifically adjusted, suggesting that their contribution to the crop type aspect was less dominant, we observed the highest N_2_O peaks in summer crops during periods of low vegetation growth—both at the start of the crop season and at the start of plant dormancy when plant N-demand was minimal (Fig. 2). During these phases, excess mineral N becomes more prone to leaching or atmospheric losses. When combined with rainfall events, nitrification and denitrification is triggered, leading increased N_2_O emissions. These findings highlight the critical importance of fertilizer timing, as applying fertilizer during these low-demand phases can exacerbate N_2_O emissions. This is further supported by the RF analysis, where N fertilizer ranked relatively low in importance for explaining variations in N_2_O emissions. Similarly, a study by Ruser et al. (2001) found that although crop specific fertilization reduced N_2_O emissions, differences in soil nitrate and moisture content across crops were the main drivers of N_2_O emission variability.

We analyzed soil CO_2_ emissions during chamber closure as a proxy for belowground biomass activity and microbial activity, both contributing to soil respiration as autotrophic (belowground) and heterotrophic respiration. In our study, higher soil respiration was associated with increased N_2_O emissions, likely due to enhanced microbial denitrification activity in the soil, which was also found by Saha et al. (2021). Additionally, organic residues in the soil can provide a labile C source that may be responsible for the increase in soil respiration and increased N_2_O emissions^40^. The elevated N_2_O emissions detected towards the end of the growing season (e.g. patch 89, Sunflower 2022; Fig. 2) could be attributed to the decomposition of crop residues during that period, which is additionally enhanced by respective rain events, increasing soil moisture contents.

Other studies^41–43^ employing RF to analyze the factors influencing agricultural N_2_O emissions identified soil (i.e. volumetric soil moisture, average daily soil temperature) and environmental conditions (i.e. rainfall, air temperature) as the most dominant predictors, which partially contrasts with our study (Fig. 3c). However, these studies either focused on fields cultivated with a single crop, were limited to a single year, or did not include crop type as a factor in the RF analysis. In contrast, our study expands on this by including multiple crops, a multi-year approach, and a comprehensive assessment of spatial variability at both the small and larger scales across different management practices. Consistent with Philibert et al. (2013), who identified crop type as one of the most important factors explaining N_2_O emissions, our results further substantiate the importance of explicitly accounting for crop diversity in N_2_O emission models.

## Conclusion

We investigated N_2_O fluxes along a transect in six agriculturally managed patches, differing in soil texture, yield potential, and crop rotation, over a 2-year period that spanned multiple crop cycles and varying weather conditions. By employing a distinct research design, we were able to analyze both fine-scale and patch-level heterogeneities in N_2_O emissions. Our results demonstrate that N_2_O emissions are shaped by the combined effects of small-scale soil texture variations and broader patch-level factors such as crop type and management.

Sandy soils exhibited pronounced microplot-scale variability in N_2_O fluxes, driven by high macro porosity and low water retention, while patch-level emissions displayed even greater heterogeneity (CV of 87%) due to differences in crop type and management practices. Hot moments, such as those triggered by fertilization and heavy rainfall, contributed significantly to total crop N_2_O balances, with shares as high as 71%. The timing and magnitude of these events varied depending on site-specific soil conditions and management factors, emphasizing the critical role of spatial and temporal dynamics in driving N_2_O emissions.

Our study provides valuable insights by linking baseline emission levels, crop-specific dynamics, and the contribution of hot moments. These relationships highlight the need for integrating spatial heterogeneity and temporal variability into predictive process-based models. By accounting for key drivers such as WFPS (%), soil respiration, and crop type, such models can better represent the multifaceted nature of N_2_O fluxes and improve the accuracy of emission estimates.

As sandy soils account for approximately 13% of global agricultural land, these findings are particularly relevant in the context of climate change, which is expected to exacerbate the frequency and intensity of droughts and heavy rainfall events^45^.

By bridging the gap between small-scale variability, driven by intrinsic field variability in soil texture and other environmental conditions, and patch-level heterogeneity, driven by differences in management, our results advance the understanding of N_2_O emissions and their entire variability spectrum in diversified agricultural systems. This knowledge supports the development of adaptive, site-specific mitigation strategies, such as optimizing fertilization timing and selecting crops better suited to specific soil conditions to minimize N_2_O emissions. At the small scale, adaptive management based on real-time environmental monitoring could reduce emissions, while at the field scale, optimized fertilization practices are crucial. Agricultural stakeholders should consider precision farming approaches to minimize emissions while maintaining productivity by using crops with high N use efficiency. We urge researchers, policymakers, and agricultural stakeholders to integrate these insights into targeted mitigation strategies that balance productivity with environmental sustainability.

## Materials and methods

### Study area and experimental setup

**Fig. 6:**
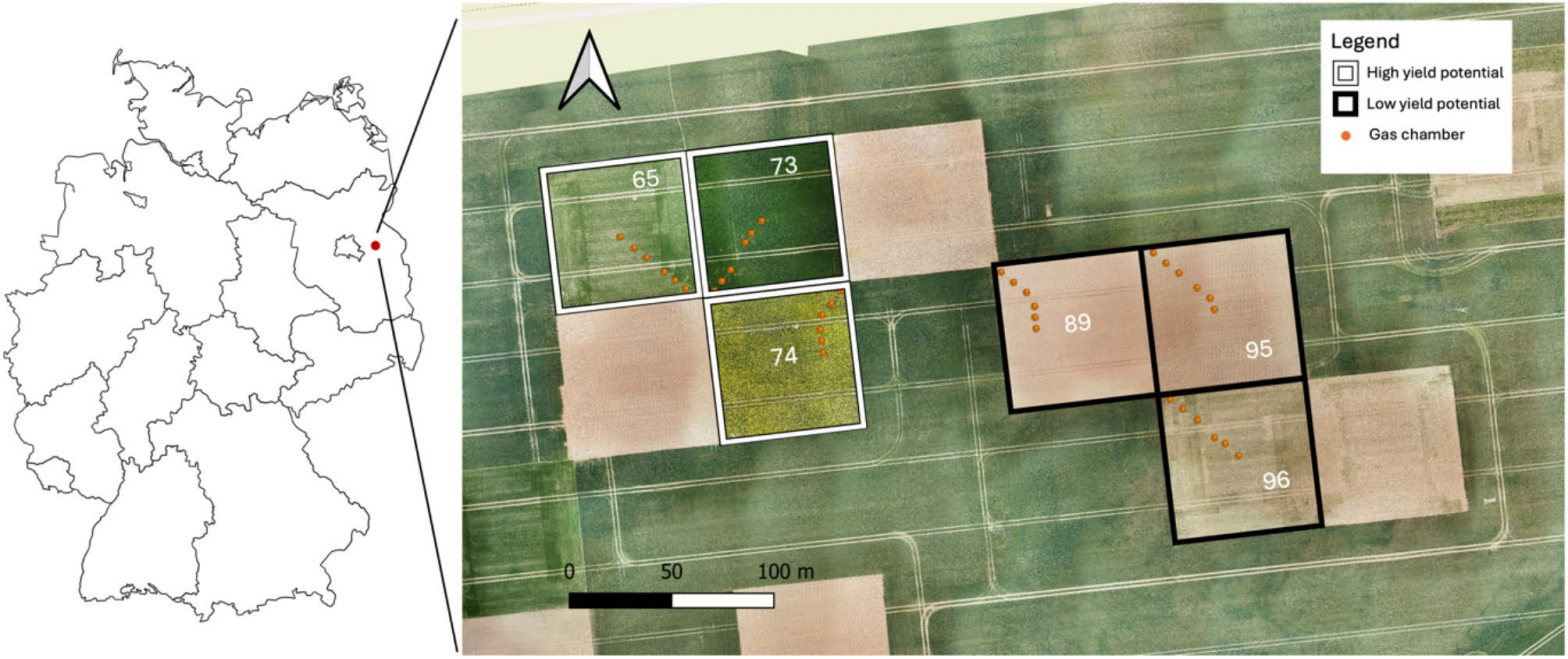
Field site consisting of three high yield (white boarder) and three low yield (black boarder) patches, with six gas chambers each, at the “patchCROP” experimental area near the village of Tempelberg, Brandenburg, NE Germany (52° 44′ 86.2″ N, 14° 14′ 05.1″ E). Imagery from May 2022, provided by L. Richter.

The study area was part of the “patchCROP” experimental field (52°44′ 86.2″ N, 14° 14′ 05.1″ E)^25^ initiated by the Leibniz Centre for Agricultural Landscape Research and consisted of six patches: three high yield potential patches (patch IDs 65, 73 and 74) and three low yield potential patches (patch IDs 89, 95 and 96) with an individual size of 0.52 ha (72 × 72 m) (Fig. 6). Yield potential zones were based on an automated cluster analysis using yield maps, soil value number, soil organic matter content and electrical resistance in the topsoil layer (0–25 cm; Donat et al., 2022). Each patch included six approximately equidistant measurement points, also referred to as microplots, which were arranged along a ~ 30 m transect within each patch, covering the maximum range of soil texture. Patch 74 (high yield) and 89 (low yield) were installed with bent transects, as the soil gradient changed in the respective direction. The temperate climate of the study area is characterized by a mean annual air temperature of 9.9 °C and mean annual precipitation of 509 mm (ZALF weather station, 2010–2024^28^). Soils were formed during the last glacial period and are part of a hummocky ground moraine landscape and can be classified as Luvisol, according to IUSS Working group WRB, 2022^47^. Due to a combination of glacial and interglacial processes, forming with erosion, soils in this area are highly heterogeneous^48^. Patches were conventionally managed, including soil tillage and pesticide applications. The specific crop rotation and patch management for each of the six patches throughout the study period from September 2021 until October 2023 is displayed in table S1.

### N_2_O flux measurements

N_2_O fluxes were measured using a non-flow-through non-steady-state (NFT-NSS) manual closed chamber system^49^. Two sets of six chambers (vol: 0.076/0.064 m^3^; area: 0.193/0.204 m^2^) consisting of white or light grey PVC, equipped with a pressure vent^50^ and one port at the top to enable air sampling with pre-evacuated glass bottles (vol: 60 ml) were used. Gas measurements were performed for six microplots per patch by setting the chambers on a PVC frame, which was inserted 5 cm deep into the soil at the beginning of each crop growth period. Chambers were sealed using a rubber band and additional elastic belts which were fixed to the frame to assure airtight closure and prevent leakage^51^. Gas samples were taken every 20 min to a total deployment time of 60 min, resulting in a four-point flux measurement. Gas samples were analyzed for N_2_O and CO_2_ concentrations using a gas chromatograph (GC-14A and GC-14B, Shimadzu Scientific Instruments, Japan), coupled with an electron capture detector (Loftpatch et al., 1997). Over each crop period, N_2_O flux measurements were carried out every 2–3 weeks. To minimize the effects of diurnal variation, measurements were consistently conducted in the morning between 8:00 AM and 12:00 PM whenever logistically feasible^53^. After fertilization and heavy rain events, measurement frequency was increased up to seven days after fertilization to cover those events more accurately, prioritizing accurate event coverage over maintaining a consistent time of day^54,55^.

### Auxiliary measurements

In addition to CO_2_ and N_2_O flux measurements, environmental variables such as volumetric soil moisture and soil and air temperature were recorded in a 15 min interval throughout the measurement period for each microplot using microclimate loggers (TMS-4, TOMST, Czech Republic), placed next to each microplot (n = 36). Soil texture analysis for every measurement point (n = 36) was carried out by the soil laboratory of the FAU Erlangen-Nürnberg. Sand fractions were determined according to DIN 19683: 1973, while the smaller particles were analyzed with a Sedigraph 5100 (Micromeritics). Soil total carbon (TC) and total nitrogen (TN) were analyzed by the central laboratory of ZALF according to ISO 10694 with a multiphase determinator (RC 612, Leco Instruments GmbH, Mönchengladbach, Germany). Soil pH was determined according to DIN ISO 10390 with an 855 Robotic Titrosampler (Deutsche Metrohm GmbH &Co. KG). Air temperature in 2 m height, wind speed and direction, relative humidity, air pressure, global radiation, and precipitation were recorded in an hourly interval by a weather station of ZALF, near the experimental field.

### Data processing and statistical analyses

The reliability of N_2_O concentration measurements during opaque chamber closure was verified prior to N_2_O flux calculation using the anticipated increase in CO_2_ concentration (from ecosystem respiration) within an opaque chamber as a quality control criterion^56^. If CO_2_ concentrations decreased over the measurement period, those measurements were deemed biased and excluded from further analysis. Outliers in the concentration data were handled using a multiple of the inter-quartile range (6 × IQR) of the regression residues as a threshold criterion. N_2_O fluxes were interpolated linearly between campaigns to obtain cropping period N_2_O emission estimates.

Calculation of N_2_O and CO_2_ fluxes (F; µmol m^−2^ s^−1^) was done according to the ideal gas law (Eq. 1) and based on the assumption of a linear concentration increase during chamber closure^57,58^.

1 $$F = \frac{pV}{{RTA}}* \Delta c/\Delta t$$

where p represents ambient air pressure (Pa), V denotes chamber volume (m^3^), R the gas constant (8.314 m^3^ Pa/K mol^−1^), T the air temperature (K), A the chamber basal area and dc/dt denotes the N_2_O or CO_2_ concentration change in the chamber headspace over the measurement time. An adaptation of the modular R script for GHG flux calculation by Vaidya et al. (2023) was used.

We identified hot moments as time periods after fertilization (14 days) and rain events (7 days).

To investigate the importance of variables explaining the mean daily N_2_O fluxes we used the random forest (RF) machine learning technique in R (V. 4.2.2 (2022-10-31); R Core Team, 2021) with the package ‘randomForest’^61^. The RF constructs an ensemble of classification or regression trees^62^, which does not require precise information about the form of the relationship between response and input variables. Autocorrelation of the data was checked with the Durbin-Watson-Test (DW) (RF model: DW = 2.15, *p* value = 0.76) to ensure reasonable results of the RF model. The DW test calculates the residues of the regression model (RF-model) and compares the sum of the squared differences of successive residuals to the sum of the squared residuals. Specific soil variables that did not improve the model were first identified using the Boruta algorithm and excluded through further refinement with the VSURF package in R. Homogeneity of variance was checked using Levene’s test for each patch, to verify variations in N_2_O emissions within and between the patches.

To analyze the interaction between the N_2_O emission and variables in the RF model, Accumulated Local Effects (ALE) plots were created. We opted for ALE over partial dependence plots (PDP) because ALE accounts for feature correlations, reducing bias. The ALE describe how features influence the prediction of a machine learning model on average^63^.

Coefficient of variation (CV) was calculated to investigate the spatial variability in-between and between the microplot of each patch. It is calculated as the ratio of the standard deviation to the mean. Higher values indicate greater variation.

2 $$CV = \frac{sd}{{\overline{x}}}*100$$

where sd is the standard deviation and $\overline{\text{x} }$ the mean of each flux calculated from the chamber concentrations.

We calculated the SC:S ratio, defined as the ratio of silt plus clay to sand content (%) of the specific soil sample to simplify the texture variable for the RF model. This provides a comprehensive soil texture metric without separating each texture component.

The slope for individual transects was calculated by subtracting the lowest elevation position (meter above sea level) from the highest and dividing by the distance (m) between the two points. To express the slope as percentage, the result was multiplied by 100.

Water filled pore space (WFPS) of the different soils was calculated from soil bulk density (BD) and the standard porosity value of 2.65 g/m^3^ with the following equation:

3 $$WFPS\,(\% ) = \frac{\theta{v}}{1 - BD/2.65}* 100$$

where θv is the volumetric water content of the specific soil sample.

## Electronic supplementary material

Below is the link to the electronic supplementary material.

Supplementary Material 1

## Data Availability

Data supporting this research are publicly available at the BONARES repository at 10.4228/zalf-akz5-yp62
