# Supplementary material for: How scale affects N2O emissions in heterogeneous fields of a diversified agricultural landscape
Source: Sci Rep. 2025 Mar 31;15:11013. doi: 10.1038/s41598-025-95630-6 (PMC11958750; doi:10.1038/s41598-025-95630-6)
Supplement: Supplementary file 1 — Supplementary Material 1 [file 41598_2025_95630_MOESM1_ESM.docx]

**Supplementary to the MS “How scale affects N_2_O emissions in heterogeneous fields of a diversified agricultural landscape”**

Table S1: Management practices of the 6 patches.

| **Field** | **Crop** | **Variety** | **Field operation** | **Date** | **Type** | **Form** | **Amount** |
| --- | --- | --- | --- | --- | --- | --- | --- |
| 65 | Winter wheat | Universum | Tillage | 12.11.21 | Cultivation |  |  |
|  |  |  | Seeding | 15.11.21 |  |  |  |
|  |  |  | Fertilization | 11.03.22 | Mineral fertilization | liquid | 80.0 kg N ha^-1^ |
|  |  |  |  | 05.04.22 | Mineral fertilization | liquid | 44.2 kg N ha^-1^ |
|  |  |  |  | 19.05.22 | Mineral fertilization | liquid | 55.1 kg N ha^-1^ |
|  |  |  | Harvest | 20.07.22 |  |  |  |
|  | Rapeseed | Ambassador | Tillage | 20.07.22 |  |  |  |
|  |  |  | Fertilization | 16.08.22 | Organic fertilization | liquid | 46.1 kg N ha^-1^ |
|  |  |  | Tillage | 29.08.22 | Seedbed preparation |  |  |
|  |  |  | Seeding | 29.08.22 |  |  |  |
|  |  |  | Fertilization | 03.03.23 | Mineral fertilization | liquid | 50.2 kg N ha^-1^ |
|  |  |  |  | 28.03.23 | Mineral fertilization | liquid | 59.2 kg N ha^-1^ |
|  |  |  | Harvest | 24.08.23 |  |  |  |
| 73 | Barley | Wallace | Tillage | 15.09.21 | Seedbed preparation |  |  |
|  |  |  | Seeding | 20.09.21 |  |  |  |
|  |  |  | Fertilization | 11.03.22 | Mineral fertilization | liquid | 44.9 kg N ha^-1^ |
|  |  |  |  | 05.04.22 | Mineral fertilization | liquid | 44.8 kg N ha^-1^ |
|  |  |  |  | 10.05.22 | Mineral fertilization | solid | 41.7 kg N ha^-1^ |
|  |  |  | Harvest | 05.07.22 |  |  |  |
|  | CC | Stala | Tillage | 18.07.22 |  |  |  |
|  |  |  | Fertilization | 16.08.22 | Organic fertilization | liquid | 38.4 kg N ha^-1^ |
|  |  |  | Tillage | 18.08.22 | Seedbed preparation |  |  |
|  |  |  | Seeding | 18.08.22 |  |  |  |
|  | Soy | Acardia | Tillage | 12.04.23 | Seedbed preparation |  |  |
|  |  |  | Seeding | 11.05.23 |  |  |  |
|  |  |  | Harvest | 28.09.23 |  |  |  |
| 74 | Rapeseed | Ambassador | Tillage | 29.07.21 | Stubble breaking |  |  |
|  |  |  | Fertilization | 10.08.21 | Organic fertilization | liquid | 41.6 kg N ha^-1^ |
|  |  |  | Tillage | 25.08.21 | Seedbed preparation |  |  |
|  |  |  | Seeding | 25.08.21 |  |  |  |
|  |  |  | Fertilization | 11.03.22 | Mineral fertilization | liquid | 60.2 kg N ha^-1^ |
|  |  |  |  | 21.03.22 | Mineral fertilization | liquid | 46.4 kg N ha^-1^ |
|  |  |  | Harvest | 20.07.22 |  |  |  |
|  | Fallow |  | Tillage | 27.07.22 |  |  |  |
|  |  |  |  | 16.08.22 |  |  |  |
|  | Barley | Wallace | Tillage | 21.09.22 | Seedbed preparation |  |  |
|  |  |  | Seeding | 21.09.22 |  |  |  |
|  |  |  | Fertilization | 03.03.23 | Mineral fertilization | liquid | 45.1 kg N ha^-1^ |
|  |  |  |  | 06.04.23 | Mineral fertilization | liquid | 66.0 kg N ha^-1^ |
|  |  |  |  | 09.05.23 | Mineral fertilization | liquid | 31.8 kg N ha^-1^ |
|  |  |  | Harvest | 07.07.23 |  |  |  |
| 89 | Sunflower | Seabird | Tillage | 25.03.22 | Seedbed preparation |  |  |
|  |  |  | Seeding | 31.03.22 |  |  |  |
|  |  |  | Fertilization | 31.03.22 | Underground fertilization | solid | 18.0 kg N ha^-1^ |
|  |  |  |  | 05.04.22 | Mineral fertilization | liquid | 54.0 kg N ha^-1^ |
|  |  |  | Harvest | 07.09.22 |  |  |  |
|  | Winter oat | Fleuron | Tillage | 29.09.22 | Seedbed preparation |  |  |
|  |  |  | Seeding | 29.09.22 |  |  |  |
|  |  |  | Fertilization | 03.03.23 | Mineral fertilization | liquid | 40.5 kg N ha^-1^ |
|  |  |  | Tillage | 06.04.23 | Breaking |  |  |
|  |  |  |  | 06.04.23 | Seedbed preparation |  |  |
|  | Summer oat | Delfin | Seeding | 06.04.23 |  |  |  |
|  |  |  | Fertilization | 25.05.23 | Mineral fertilization | solid | 30.2 kg N ha^-1^ |
|  |  |  | Harvest | 24.08.23 |  |  |  |
| 95 | Maize | P 8329 | Tillage | 27.04.22 | Subsoiling |  |  |
|  |  |  | Seeding | 29.04.22 |  |  |  |
|  |  |  | Fertilization | 29.04.22 | Underground fertilization | solid | 18.0 kg N ha^-1^ |
|  |  |  |  | 20.05.22 | Mineral fertilization | solid | 64.8 kg N ha^-1^ |
|  |  |  |  | 23.06.22 | Mineral fertilization | solid | 45.1 kg N ha^-1^ |
|  |  |  | Harvest | 20.10.22 |  |  |  |
|  | Fallow |  | Tillage | 21.10.22 |  |  |  |
|  |  |  |  | 14.11.22 |  |  |  |
|  | Lupine | Boregine | Tillage | 20.03.23 | Seedbed preparation |  |  |
|  |  |  | Seeding | 20.03.23 |  |  |  |
|  |  |  | Harvest | 13.07.23 |  |  |  |
| 96 | Winter oat | Fleuron | Tillage | 06.10.21 | Seedbed preparation |  |  |
|  |  |  | Seeding | 06.10.21 |  |  |  |
|  |  |  | Tillage | 10.03.22 | Breaking |  |  |
|  |  |  |  | 10.03.22 | Seedbed preparation |  |  |
|  | Summer oat | Delfin | Seeding | 18.03.22 |  |  |  |
|  |  |  | Fertilization | 06.04.22 | Mineral fertilization | liquid | 75.7 kg N ha^-1^ |
|  |  |  | Harvest | 15.07.22 |  |  |  |
|  | CC | Stala | Tillage | 18.07.22 |  |  |  |
|  |  |  | Fertilization | 16.08.22 | Organic fertilization | liquid | 38.4 kg N ha^-1^ |
|  |  |  | Tillage | 18.08.22 | Seedbed preparation |  |  |
|  |  |  | Seeding | 18.08.22 |  |  |  |
|  | Maize | P 8329 | Tillage | 09.05.23 | Seedbed preparation |  |  |
|  |  |  | Seeding | 09.05.23 |  |  |  |
|  |  |  | Fertilization | 09.05.23 | Underground fertilization | solid | 13.6 kg N ha^-1^ |
|  |  |  |  | 25.05.23 | Mineral fertilization | solid | 56.8 kg N ha^-1^ |
|  |  |  |  | 22.06.23 | Mineral fertilization | solid | 35.5 kg N ha^-1^ |
|  |  |  | Harvest | 25.10.23 |  |  |  |

Table S2: Soil characteristics of the upper soil (0-30cm) for each microplot (MP).

|  | **Field** | **Micro plot** | **sand (%)** | **silt (%)** | **clay (%)** | **pH (-)** | **C/N (-)** | **TC (%)** | **TN (%)** |
| --- | --- | --- | --- | --- | --- | --- | --- | --- | --- |
| High yield | 65 | 1 | 70.7 | 20.9 | 8.3 | 6.4 | 12.3 | 0.96 | 0.08 |
|  |  | 2 | 72.1 | 19.6 | 8.3 | 6.0 | 12.5 | 0.96 | 0.08 |
|  |  | 3 | 72.1 | 19.8 | 8.1 | 5.8 | 12.4 | 0.94 | 0.08 |
|  |  | 4 | 69.8 | 20.9 | 9.4 | 5.8 | 11.9 | 0.99 | 0.09 |
|  |  | 5 | 69.5 | 21.5 | 9.1 | 6.1 | 12.6 | 0.94 | 0.07 |
|  |  | 6 | 72.0 | 20.3 | 7.7 | 5.7 | 12.0 | 0.84 | 0.07 |
|  | 73 | 1 | 70.2 | 23.0 | 6.8 | 6.4 | 10.7 | 1.05 | 0.10 |
|  |  | 2 | 70.6 | 24.8 | 4.6 | 6.4 | 11.3 | 0.91 | 0.08 |
|  |  | 3 | 71.4 | 23.2 | 5.4 | 6.3 | 11.3 | 0.90 | 0.08 |
|  |  | 4 | 70.8 | 22.3 | 6.9 | 6.3 | 11.2 | 0.91 | 0.08 |
|  |  | 5 | 68.4 | 23.7 | 7.9 | 6.3 | 10.9 | 0.91 | 0.08 |
|  |  | 6 | 68.8 | 23.3 | 8.0 | 6.4 | 11.0 | 0.93 | 0.08 |
|  | 74 | 1 | 68.7 | 24.4 | 6.9 | 6.2 | 10.5 | 0.79 | 0.08 |
|  |  | 2 | 68.1 | 24.2 | 7.7 | 6.2 | 10.2 | 0.72 | 0.07 |
|  |  | 3 | 68.7 | 25.4 | 5.9 | 6.2 | 10.4 | 0.84 | 0.08 |
|  |  | 4 | 69.9 | 24.1 | 5.9 | 6.2 | 10.8 | 0.95 | 0.09 |
|  |  | 5 | 67.6 | 26.0 | 6.3 | 6.0 | 10.4 | 0.86 | 0.08 |
|  |  | 6 | 68.3 | 24.1 | 7.6 | 6.1 | 10.5 | 0.87 | 0.08 |
| Low yield | 89 | 1 | 73.0 | 22.4 | 4.6 | 5.5 | 9.2 | 0.69 | 0.08 |
|  |  | 2 | 76.3 | 19.0 | 4.6 | 5.8 | 9.4 | 0.59 | 0.06 |
|  |  | 3 | 77.0 | 17.7 | 5.3 | 5.5 | 8.7 | 0.63 | 0.07 |
|  |  | 4 | 77.0 | 18.8 | 4.2 | 5.7 | 9.5 | 0.65 | 0.07 |
|  |  | 5 | 80.9 | 14.9 | 4.1 | 5.5 | 9.3 | 0.73 | 0.08 |
|  |  | 6 | 81.8 | 14.6 | 3.6 | 6.1 | 9.9 | 0.67 | 0.07 |
|  | 95 | 1 | 81.8 | 13.3 | 5.0 | 6.4 | 10.2 | 0.68 | 0.07 |
|  |  | 2 | 84.2 | 11.2 | 4.6 | 6.0 | 10.2 | 0.66 | 0.07 |
|  |  | 3 | 84.0 | 11.3 | 4.7 | 5.9 | 10.3 | 0.67 | 0.07 |
|  |  | 4 | 84.5 | 10.7 | 4.7 | 6.0 | 9.3 | 0.67 | 0.07 |
|  |  | 5 | 83.2 | 11.8 | 5.0 | 5.5 | 10.1 | 0.70 | 0.07 |
|  |  | 6 | 81.9 | 12.3 | 5.8 | 5.8 | 9.2 | 0.64 | 0.07 |
|  | 96 | 1 | 80.1 | 14.6 | 5.3 | 5.2 | 11.1 | 0.83 | 0.07 |
|  |  | 2 | 80.1 | 14.1 | 5.8 | 5.5 | 11.6 | 0.96 | 0.08 |
|  |  | 3 | 80.7 | 14.4 | 4.9 | 5.2 | 11.1 | 0.83 | 0.08 |
|  |  | 4 | 83.6 | 13.2 | 3.3 | 5.6 | 11.0 | 0.80 | 0.07 |
|  |  | 5 | 83.3 | 12.6 | 4.0 | 5.3 | 10.3 | 0.74 | 0.07 |
|  |  | 6 | 83.3 | 12.5 | 4.1 | 5.6 | 10.8 | 0.80 | 0.07 |


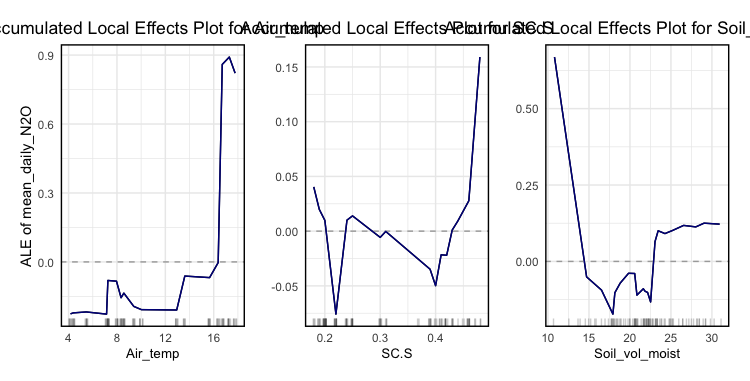


Figure S1: Accumulated Local Effects Plots (ALE) for variables defining N_2_O emissions.

Table S3: Hot moments: contribution of fertilization and rain events to the total crop N_2_O-N emissions for each patch.

|  | **crop 1** | | **crop 2** | | **crop 3** | | **crop 4** | |
| --- | --- | --- | --- | --- | --- | --- | --- | --- |
| **Patch** | **g N_2_O-N ha^-1^ crop^-1^** | **% of total** | **g N_2_O-N ha^-1^ crop^-1^** | **% of total** | **g N_2_O-N ha^-1^ crop^-1^** | **% of total** | **g N_2_O-N ha^-1^ crop^-1^** | **% of total** |
| 65 | 245 | 62 | 184 | 46 |  |  |  |  |
| 73 | 75 | 30 | 57 | 28 | 134 | 52 |  |  |
| 74 | 83 | 18 | 32 | 37 | 419 | 61 |  |  |
| 89 | 130 | 43 | 11 | 7 | 43 | 38 |  |  |
| 95 | 549 | 48 | 26 | 35 |  |  |  |  |
| 96 | 20 | 6 | 383 | 41 | 470 | 48 | 1638 | 71 |

**
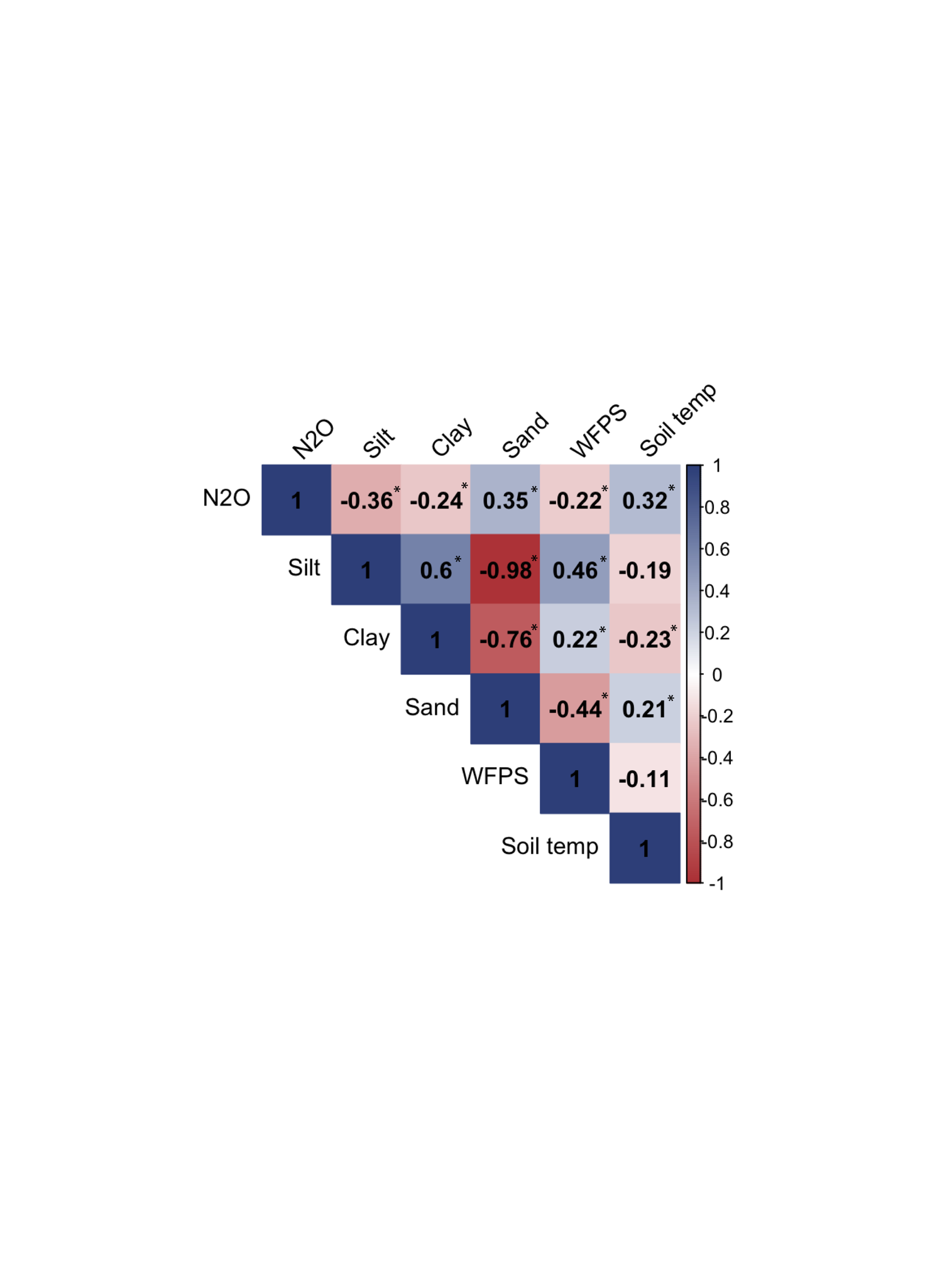
**

Figure S2: Correlation plot of mean daily N_2_O-N (g ha^-1^ crop^-1^) with selected soil environmental parameters (Silt, clay, sand content and WFPS in percentage, soil temperature in Celsius). *Significance p < 0.05.
